# Anticholinesterse and antioxidant investigations of crude extracts, subsequent fractions, saponins and flavonoids of *atriplex laciniata* L.: potential effectiveness in Alzheimer’s and other neurological disorders

**DOI:** 10.1186/s40659-015-0011-1

**Published:** 2015-04-01

**Authors:** Zul Kamal, Farhat Ullah, Muhammad Ayaz, Abdul Sadiq, Sajjad Ahmad, Anwar Zeb, Abid Hussain, Muhammad Imran

**Affiliations:** Department of Pharmacy, Shaheed Benazir Butto University, Sheringal, Dir (U), KPK Pakistan; Department of Pharmacy, University of Malakand, Chakdara, 18000 Dir (L), KPK Pakistan

**Keywords:** Phenolic & flavonoid contents, DPPH, ABTS, H_2_O_2_, Gallic acid, IC_50_ and anticholinesterase

## Abstract

**Background:**

*Atriplex laciniata* L. was investigated for phenolic, flavonoid contents, antioxidant, anticholinesterase activities, in an attempt to explore its effectiveness in Alzheimer’s and other neurological disorders. Plant crude methanolic extract (Al.MeF), subsequent fractions; n-hexane (Al.HxF), chloroform (Al.CfF), ethyl acetate (Al.EaF), aqueous (Al.WtF), Saponins (Al.SPF) and Flavonoids (Al.FLVF) were investigated for DPPH, ABTS and H_2_O_2_ free radical scavenging activities. Further these extracts were subjected to acetylcholinesterase (AChE) & butyrylcholinesterase (BChE) inhibitory activities using Ellman’s assay. Phenolic and Flavonoid contents were determined and expressed in mg Gallic acid GAE/g and Rutin RTE/g of samples respectively.

**Results:**

In DPPH free radicals scavenging assay, Al.FLVF, Al.SPF and Al.MeF showed highest activity causing 89.41 ± 0.55, 83.37 ± 0.34 and 83.37 ± 0.34% inhibition of free radicals respectively at 1 mg/mL concentration. IC_50_ for these fractions were 33, 83 and 82 μg/mL respectively. Similarly, plant extracts showed high ABTS scavenging potential, i.e. Al.FLVF (90.34 ± 0.55), Al.CfF (83.42 ± 0.57), Al.MeF (81.49 ± 0.60) with IC_50_ of 30, 190 and 70 μg/ml respectively. further, H_2_O_2_ percent scavenging was highly appraised in Al.FLVF (91.29 ± 0.53, IC_50_ 75), Al.SPF (85.35 ± 0.61, IC_50_ 70) and Al.EaF (83.48 ± 0.67, IC_50_ 270 μg/mL). All fractions exhibited concentration dependent AChE inhibitory activity as; Al.FLVF, 88.31 ± 0.57 (IC_50_ 70 μg/mL), Al.SPF, 84.36 ± 0.64 (IC_50_ 90 μg/mL), Al.MeF, 78.65 ± 0.70 (IC_50_ 280 μg/mL), Al.EaF, 77.45 ± 0.46 (IC_50_ 270 μg/mL) and Al.WtF 72.44 ± 0.58 (IC_50_ 263 μg/mL) at 1 mg/mL. Likewise the percent BChE inhibitory activity was most obvious in Al.FLVF 85.46 ± 0.62 (IC_50_ 100 μg/mL), Al.CfF 83.49 ± 0.46 (IC_50_ 160 μg/mL), Al.MeF 82.68 ± 0.60 (IC_50_ 220 μg/mL) and Al.SPF 80.37 ± 0.54 (IC_50_ 120 μg/mL).

**Conclusions:**

These results stipulate that *A. laciniata* is enriched with phenolic and flavonoid contents that possess significant antioxidant and anticholinestrase effects. This provide pharmacological basis for the presence of compounds that may be effective in Alzheimer’s and other neurological disorders.

## Background

Free radical especially reactive oxygen species (ROS), such as superoxide (O_2_^−^), hydroxyl (OH) and hydrogen peroxide (H_2_O_2_) have greater brunt on human both from within the body and from their surroundings. If the body fails to eliminate, ROS can attack on biomolecules such as lipids, proteins, enzymes, DNA and RNA. Though, human body possesses many defense mechanisms through antioxidant enzymes and non-enzymatic compounds against these oxidative stresses. But when these free radicals go out of control, the organism becomes incapable to scavenge all ROS which may lead to the development of chronic diseases, such as cancer, arteriosclerosis, nephritis, diabetes mellitus, liver injury, rheumatism, ischemia, cardiovascular and neurodegenerative disorders such as Alzheimer’s and Parkinson’s disease [1]. Chemical agents, radiation, toxins, deep fried foods and environmental factors such as pollution, radiation, cigarette smoke and herbicides can generate these reactive free radicals [2].

Among the neurological disorders, Alzheimer’s disease (AD) is one of indefinite neurodegenerative diseases and is the most common cause of dementia [3]. AD is characterized by deposition of amyloid plaque having insoluble protein fragments, Amyloid Beta (Aβ), and by manifestation of neurofibrillary tangles in the region of hippocampus and cortex [4]. AD is also associated with loss of synapses, synaptic dysfunctions, mitochondrial abnormalities and inflammatory stress [5] that may lead to multi neurotransmitters deficiency like, acetylcholine [6], noradrenalin [7], serotonin [8] and somatostatin [9]. Acetylcholine (neurotransmitter) is involved in the signal transmission in the synapse and its pharmacological action is terminated primarily by AChE and secondly by BChE [10]. Therefore, inhibitors of these metabolizing enzymes have become important alternatives in the treatment of AD [11]. Federal Drug Administration (FDA) have approved Tacrine, Rivastigmine, Donepezil, for symptomatic treatment, but these drugs do not impede the progression of disease and are associated with various adverse effects [12]. Besides these, anti inflammatory drugs [13], antioxidants [14], calcium channel blockers [15], cholesterol lowering drugs and metal chelating agents [16] possess a protective role in AD. Medicinal plants used in the form of crude drugs, spices and vegetables have been widely used in Chinese and Ayurvedic cultures for many centuries to restore declining cognitive functions [17]. Several plants are have been identified for its role in dementia such as *Curcuma longa*, *Zingiber officinale* [18], *Huperzia serrata* (toothed clubmoss) [19], *Ginkgo Biloba* [20]*, Catharanthus Roseus* [21], *Panax ginseng* [22]*,* and *Galanthus nivalis;* which contains Galanthamine, an anticholinestrase alkaloid has been recently approved for the treatment of AD [23]. Scientists have shown interest to treat and prevent AD with herbs, spices and vegetables [24]. The medicinal plants can produce the therapeutic effect by producing antiamyloid effect, antioxidant effect, antiapoptotic effect, enhancing synaptic functions, inhibiting cholinesterase and N-Methyl D-Aspartate (NMDA) receptors [25]. Herbal drugs have dominance over synthetic drug because they show less toxicity, easily cross blood brain barrier and exert multiple synergistic effects [24]. Medicinal plants used traditionally have been improved cholinergic and cognitive functions by acting as cholinesterase inhibitors *in-vitro* as well as *in-vivo* [23,26]. Thus evaluating these enzymes inhibitory and antioxidants effects of natural products in vitro may be used as treatment option in AD and other cognitive dysfunctions [24].

Similarly phenols and flavonoids are important plant secondary metabolites, that’s having conjugated ring structures and hydroxyl groups, that may have the potential to function as antioxidants by scavenging the free radicals which are involved in oxidative processes via hydrogenation or complexation with oxidizing species and may resist many oxidative stresses and diseases [2]. The incessant formation of free radicals in human’s body can be controlled naturally and synthetically by different valuable compounds known as antioxidants. Presently butylated hydroxy toluene (BHT), butylated hydroxy anisole (BHA), gallic acid esters and tertiary butylated hydroquinon available synthetic antioxidants, but their use is restricted and there is a propensity to surrogate them with natural antioxidants that may be found in medicinal plants [27]. Several reports on the antioxidant and radical-scavenging activities of crude extracts and pure natural compounds have been reported [28,29].

*A. laciniata* (Chenopodiaceae), a halophyte, one of the salt bushes species of Atriplex. Commonly known by their English name cut-leaf saltbush, Belgian orach, four wings saltbush, frosted orache, frosted sea orach. The plant is native to Australia, North America, South America and Eurasia [30]. Ethno botanists have not indicated any traditional use of this plant in Pakistan. Though, no toxicological or pharmacological studies have been carried out in detail so far on this specie. However antimicrobial, antineoplastic, analgesic, antipyretic, anthelmintic, insecticidals, cytotoxic, antioxidants, anticholinesterase, gastro and hepatotoxic, antiparasites, laxative, sedative activities have already been reported in various species of Chenopodiaceae [31,32]. Research has been paying attention on the biological effects of plants which are traditionally used as cholinesterase inhibitors and radical scavenging *in-vitro* as well as *in-vivo* [33]. This study is also focused on preliminary anticholinestrase and antioxidant potential of *A. laciniata.*

## Results

### Total phenolic and flavonoid content

The extraction yield of phenolics (mg GAE/g of sample) and flavonoids (mg RTE/ g of sample) contents in different fractions of *A. laciniata* are presented in Table 1. Al.MeF, Al.EaF and Al.CfF fractions exhibited high phenolic contents. Total phenolics were, 311.32 ± 0.54 (Al.MeF), 152.59 ± 0.48 (Al.HxF), 231.12 ± 0.58 (Al.CfF), 278.79 ± 0.45 (Al.EaF) and 190.62 ± 0.62 (Al.WtF) mg GAE/g of sample. Whereas, total flavonoids (mg RTE/g of sample) were 144.37 ± 0.52 (Al.MeF), 14.38 ± 0.51 (Al.HxF), 160.41 ± 0.55 (Al.CfF), 52.33 ± 0.46 (Al.EaF) & 82.25 ± 0.55 (Al.WtF).The concentration of phenolics among different fractions were in an ascending order of Al.MeF > Al.EaF > Al.CfF > Al.WtF > Al.HxF. Similarly Al.CfF and Al.MeF fractions exhibited highest flavonoid contents. The ascending order for total flavonoids contents were Al.CfF > Al.MeF > Al.Wtf > Al.EaF > Al.HxF.

**Table 1:** **Total phenolics and flavonoids contents of crude methanolic and their subsequent fractions of** ***Atriplex laciniata***

**Samples**	**Total phenolics (mg GAE/g of sample)**	**Total flavonoids (mg RTE/g of sample)**
**Al.MeF**	311.32 ± 0.54	144.37 ± 0.52
**Al.HxF**	152.59 ± 0.48	14.38 ± 0.51
**Al.CfF**	231.12 ± 0.58	160.41 ± 0.55
**Al.EaF**	278.79 ± 0.45	52.33 ± 0.46
**Al.WtF**	190.62 ± 0.62	82.25 ± 0.55

GAE = galic acid equivalent, RTE = rutin equivalent, each value in the table is represented as mean ± SEM (n = 3).

### DPPH radical scavenging effect

Comparatively the most stable organic radical DPPH is extensively used in modeling systems to explore the scavenging activities of several natural compounds, such as phenolics, flavonoids, as well as crude mixtures, such as methanolic, *n*-hexane, ethyl acetate or water extracts from plants. The antioxidants scavenged the DPPH radical through the donation of electrons forming the reduced DPPH, upon which color changes from purple to yellow. Table 2 shows concentration dependent DPPH percent inhibitions and IC_50_ values for radical scavenging activities of crude saponins, flavonoids and different extract fractions of the *A. laciniata.* Among the tested fractions In DPPH radical scavenging evaluation Al.FLVF (IC_50_ 33 μg/mL), Al.SPF (IC_50_ 83 μg/mL) and Al.MeF (IC_50_ 82 μg/mL) shows highest percent inhibitions in concentration dependent manner that may be attributed due to their high phenolic and flavonoid contents. The DPPH free radical scavenging potential of the tested fractions were in an ascending order Al.FLVF > Al.SPF > Al.MeF > Al.CfF > Al.EaF > Al.Wtf > Al.HxF. In Al.FLVF the difference in P (>0.O5) value were statistically non significant in comparison with positive control at highest concentrations.

**Table 2:** **Results of antioxidant potential of** ***Atriplex laciniata*** **against DPPH and ABTS free Radicals using ascorbic acid as standard**

**Samples**	**Conc. (μ** **g/mL)**	**DPPH Percent inhibition**	**DPPH IC**_**50**_ **(μ** **g/mL)**	**ABTS Percent inhibition**	**ABTS IC**_**50**_ **(μ** **g/mL)**
Al.MeF	1000	83.23 ± 0.44^**^	82	81.49 ± 0.60^***^	70
	500	79.50 ± 0.61 ^ns^		75.45 ± 0.59^**^	
	250	68.47 ± 0.46^***^		64.52 ± 0.60^***^	
	125	53.47 ± 0.70^***^		57.58 ± 0.57^***^	
Al.HxF	1000	65.57 ± 0.43^***^	270	69.90 ± 1.04 ^***^	215
	500	58.12 ± 0.94^***^		61.53 ± 0.65^***^	
	250	48.76 ± 1.09^***^		52.20 ± 1.04^***^	
	125	34.38 ± 0.50^***^		40.50 ± 0.73^***^	
Al.CfF	1000	74.50 ± 0.56^***^	210	83.42 ± 0.57^***^	190
	500	61.40 ± 0.55^***^		72.32 ± 0.52^***^	
	250	52.36 ± 0.57^***^		58.27 ± 0.57^***^	
	125	43.30 ± 0.52^***^		37.30 ± 0.64^***^	
Al.EaF	1000	69.37 ± 0.58^***^	382	76.34 ± 0.63^***^	360
	500	62.29 ± 0.43^***^		67.54 ± 0.46^***^	
	250	24.58 ± 0.56^***^		33.48 ± 0.60^***^	
	125	17.39 ± 0.60^***^		28.52 ± 0.66^***^	
Al.WtF	1000	67.83 ± 1.07^***^	210	69.37 ± 1.10^***^	310
	500	61.56 ± 0.69^***^		57.56 ± 0.45^***^	
	250	52.48 ± 0.54^***^		46.36 ± 0.55^***^	
	125	40.90 ± 1.16^***^		35.57 ± 0.84^***^	
Al.SPF	1000	83.37 ± 0.52^**^	83	78.40 ± 0.51^***^	92
	500	73.38 ± 0.76^***^		71.44 ± 0.55^***^	
	250	67.24 ± 0.80^***^		63.47 ± 0.52^***^	
	125	54.40 ± 0.76^***^		52.39 ± 0.49^***^	
Al.FLVF	1000	89.41 ± 0.55 ^ns^	33	90.34 ± 0.55 ^ns^	30
	500	83.27 ± 0.58 ^ns^		86.36 ± 0.49^***^	
	250	76.35 ± 0.51 ^ns^		73.52 ± 0.62 ^ns^	
	125	63.28 ± 0.57 ^*^		67.42 ± 0.55 ^ns^	
Ascorbic Acid	1000	87.90 ± 0.96	14	89.30 ± 0.61	37
	500	83.08 ± 0.47		80.37 ± 0.54	
	250	79.85 ± 2.24		73.37 ± 0.64	
	125	67.36 ± 0.57		64.45 ± 0.65	

Values significantly different as compared to positive control, *:P < 0.05, **:P < 0.01, ***:P < 0.001, ns: P >0.05. Values expressed as Percent inhibition (Mean ± SEM of n = 3) and IC_50_.

### ABTS free radical scavenging assay

Crude extracts of *A. laciniata* exhibited high activity against ABTS free radicals at various concentrations that was comparable with DPPH scavenging, as summarized in Table 2. Among different fractions, Al.FLVF was most potent (IC_50_ 30 μg/mL), followed by Al.CfF, Al.MeF and Al.SPF with IC_50_ of 190, 70 and 92 μg/mL respectively. The ABTS percent inhibitions were compared with the positive control ascorbic acid (IC_50_ 37 μg/mL).

### Hydrogen peroxide scavenging activity

**Figure 1:**
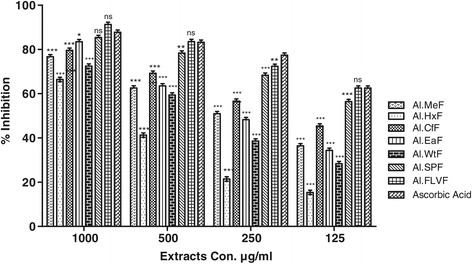
**Antioxidant assay of plant extracts using H**_**2**_ **0**_**2**_ **assay.** Values represent percent radical scavenging (mean ± SEM) of three replicates. Values significantly different as compare to positive control *:P < 0.05, **:P < 0.01 and ***:P < 0.001.

**Figure 2:**
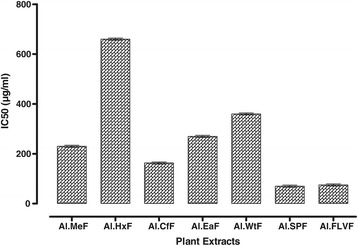
**IC**_**50**_ **values For antioxidant activity of Plant extracts using H**_**2**_ **O**_**2**_ **assay.**

Results of H_2_O_2_ percent scavenging are summarized in Figure 1, IC_50_ values are given in Figure 2. Al.FLVF (91.29 ± 0.53) displayed greater H_2_O_2_ percent scavenging than the standard ascorbic acid (87.76 ± 0.60) at highest concentration. Similarly Al.SPF (85.35 ± 0.61) and Al.EaF (83.48 ± 0.67) shows comparable results, that reflects the presence of antioxidant potential in *A. laciniata.*

### Correlation between antioxidant, total phenolic and flavonoid contents

**Figure 3:**
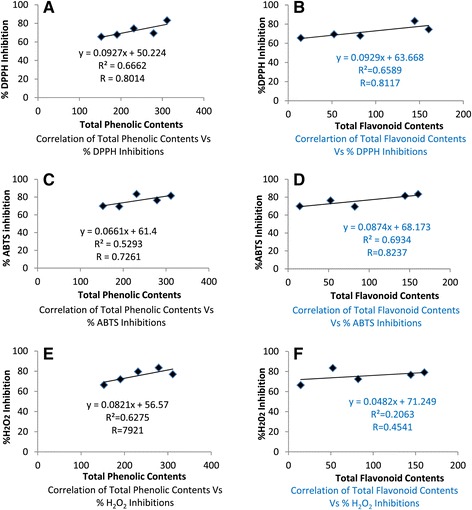
**Correlation coefficients (R** ^**2**^ **) of antioxidant activity, for total phenolic vs DPPH** **(A)** **, total phenolic vs ABTS** **(C)** **, total phenolic vs H**_**2**_ **O**_**2**_ **(E)** **and flavonoid contents Vs DPPH** **(B)** **, flavonoid contents Vs ABTS** **(D)** **and flavonoid contents Vs H**_**2**_ **O**_**2**_ **(F)** **in crude various fractions of** ***Atriplex laciniata*** **L.**

The correlation coefficients (R^2^) of antioxidant activity (DPPH, ABTS and H_2_O_2_), total phenolic contents (TPC) and total flavonoid contents (TFC) of Al.MeF, Al.HxF, Al.CfF, Al.EaF and Al.WtF are shown in Figure 3. The R^2^ values of %DPPH inhibition versus TPC (Figure 3A) and TFC (Figure 3B) are 0.66 and 0.65, wherein % ABTS inhibition versus TPC (Figure 3C) and TFC (Figure 3D), that is 0.52 and 0.69 respectively, that indicate a moderate correlation between DPPH, ABTS, TPC and TFC of *A. laciniata* extracts. Likewise, R^2^ in % H_2_O_2_ inhibition versus TPC (Figure 3E) and TFC (Figure 3F) are 0.62 and 0.20 respectively. From this statistics, we determined a poor correlation between % H_2_O_2_ inhibition and TFC while moderate correlation versus TPC. Linear regression analysis showed a moderate correlation between antioxidant activity versus total phenolic and flavonoid contents. High phenolic and flavonoid content is only one of the antioxidant capacity indicators.

### Acetylcholinesterase (AChE) and butyrylcholinesterase (BChE) inhibition

Inhibition of AChE and BChE are the key enzymes in the breakdown of acetylcholine and butyrylcholine that may be considered as one of the treatment approaches against several neurological disorders such as Alzheimer’s disease, senile dementia, ataxia, and myasthenia gravis. Most of the Plants have traditionally been used to enhance a cognitive function and to alleviate other symptoms associated with Alzheimer’s disease nowadays [1]. Table 3 shows the AChE and BChE percent inhibitions and IC_50_ values of the *A. laciniata* various crude extracts using Galantamine as a positive control. Generally, the extracts exhibited a dose-dependent AChE and BChE percent inhibitions. At highest concentrations, all the crude fractions had a better AChE and BChE percent inhibition values ranging from 64.36 ± 0.61 to 88.31 ± 0.57. Al.FLVF, Al.SPF and Al.MeF showed most potent AChE IC_50_ values of 70, 90, 280 μg/mL respectively as compared to that of Galantamine 52 μg/mL. The ascending order for AChE inhibitory activity among the tested fractions were Al.FLVF > Al.SPF > Al.MeF > Al.EaF > Al.HxF > Al.Wtf > Al.CfF. Similarly Al.FLVF, Al.CfF and Al.MeF displaying the most effective BChE median inhibitory concentrations (IC_50_) of 100, 160 and 220 μg/mL respectively, while that for the positive control was 47 μg/mL. The BChE inhibitory activity among the given fractions were in rising order of Al.FLVF > Al.CfF > Al.MeF > Al.SPF > Al.Wtf > Al.EaF > Al.HxF.

**Table 3:** **AChE & BChE inhibitiory potentials of plant extracts using galanthamine as standard**

**Samples**	**Concentrations (** **μ** **g/mL)**	**Percent Inhibition (AChE)**	**AChE IC**_**50**_ **(** **μ** **g/mL)**	**Percent inhibition (BChE)**	**BChE IC**_**50**_ **(** **μ** **g/mL)**
**Al.MeF**	1000	78.65 ± 0.70^***^	280	82.68 ± 0.60^***^	220
	500	67.25 ± 0.55^***^		69.42 ± 0.60^***^	
	250	47.21 ± 0.62^***^		52.51 ± 0.66^***^	
	125	31.55 ± 0.56^***^		41.27 ± 0.62^***^	
**Al.HxF**	1000	72.51 ± 0.62^***^		64.36 ± 0.61^***^	400
	500	63.44 ± 0.44^***^	310	53.40 ± 0.52^***^	
	250	42.46 ± 0.47^***^		39.46 ± 0.60^***^	
	125	23.68 ± 0.64^***^		22.52 ± 0.49^***^	
**Al.CfF**	1000	67.42 ± 0.46 ^***^		83.49 ± 0.46^***^	160
	500	53.53 ± 0.41^***^	390	72.48 ± 0.50^***^	
	250	40.62 ± 0.69^***^		59.59 ± 0.66^***^	
	125	18.46 ± 0.67^***^		45.57 ± 0.63^***^	
**Al.EaF**	1000	77.45 ± 0.55 ^***^		74.41 ± 0.60^***^	260
	500	57.63 ± 0.64^***^	270	61.53 ± 0.71^***^	
	250	48.45 ± 0.66 ^***^		49.45 ± 0.77^***^	
	125	36.36 ± 0.50^***^		31.75 ± 0.58^***^	
**Al.WtF**	1000	72.44 ± 0.58^***^		78.62 ± 0.36^***^	210
	500	60.51 ± 0.59^***^	263	69.22 ± 0.47^***^	
	250	48.62 ± 0.70^***^		52.52 ± 0.71^***^	
	125	31.47 ± 0.55^***^		43.52 ± 0.70^***^	
**Al.SPF**	1000	84.36 ± 0.64 ^***^		80.37 ± 0.54^***^	120
	500	77.34 ± 0.65^***^	90	73.44 ± 0.50^***^	
	250	65.58 ± 0.67^***^		62.51 ± 0.72^***^	
	125	53.60 ± 0.50^***^		49.28 ± 0.61^***^	
**Al.FLVF**	1000	88.31 ± 0.57^ns^		85.46 ± 0.62^***^	100
	500	79.46 ± 0.63 ^*^	70	76.58 ± 0.69^***^	
	250	67.53 ± 0.49 ^***^		65.68 ± 0.42^***^	
	125	59.38 ± 0.50^ns^		52.46 ± 0.72^***^	
**Galanthamine**	1000	91.37 ± 0.65		94.50 ± 0.71	47
	500	83.33 ± 0.55	52	85.47 ± 0.59	
	250	73.31 ± 0.60		71.72 ± 0.51	
	125	61.32 ± 0.54		65.37 ± 0.69	

Result expressed as % inhibition (mean ± SEM of n = 3) and IC50 values. Values significantly different as compared to positive control, *:P < 0.05, ***:P < 0.001, ns: P >0.05.

## Discussion

Natural products showing their therapeutic potential and effectiveness in the treatment and management of neurodegenerative diseases such as Alzheimer’s, Parkinson and Epilepsy are getting much consideration in this modern era. There has been a continuous search for the development of novel and cost effective drugs from natural sources. The therapeutic and pharmacological properties of *A. laciniata* have not been reported in any journal before. The results of the present study suggest that this plant may be a generous source of secondary metabolites, which act as natural antioxidants and cholinesterase inhibitors.

*A. laciniata* extracts contained high levels of total phenolic and flavonoid contents. The extraction yield of TPC and TFC were reported in Table 1. Previous reports indicate that phenols and flavonoids are free radical terminators and scavengers that may be attributed due to their molecular weight, presence of aromatic rings and nature of OH group’s substitution [34,35]. In the current study, the correlation coefficient for phenolic, flavonoid contents in Al.MeF, Al.HxF, Al.CfF, Al.EaF, Al.WtF versus activities including DPPH, ABTS free radical scavenging indicates that phenols and flavonoids were the main components responsible for the antioxidant behavior of crude extracts. Similarly Al.FLVF and Al.SPF also exhibited strapping antioxidant via DPPH reduced to DPPH-H, prevention of ABTS to ABTS^++^ radical cationation and break down of peroxides. It has been found that plant metabolites such as vitamins, like E and C, carotenoids or enzymes involved in the antioxidant mechanisms, shows their biochemical effects via several mechanisms, including hindrance of chain initiation, chelation of metal ions, breakdown of peroxides, sustained hydrogen abstraction, reductive ability and radical scavenging [36]. The free radicals like hydroxyl, nitric oxide, superoxide & lipid peroxyl and non-free radicals mostly include singlet oxygen and hydrogen peroxide, can be scavenged by natural antioxidants, that may be beneficial in various physiological and neurodegenerative disorders [37]. Though in all living organism there is a protective antioxidant system that protect the body systems from the consequences of free radical formations [36].

The acetylcholinesterase enzyme is an attractive target for the rational drug design and for the discovery of mechanism based inhibitors because of its role in the hydrolysis of the neurotransmitter acetylcholine. AChE inhibitors are the most effective approach to treat the cognitive symptoms of Alzheimer disease and other possible therapeutic applications in the treatment of Parkinson.s disease, senile dementia, and ataxia, among others. Additionally the extract showed considerable anti-cholinesterase inhibition, approximately halving the breakdown of substrate by human cholinesterase. All the plant extracts contained some level of inhibitory activity against AChE and BChE. A low IC_50_ value is indicative of a good inhibition of the enzyme. Al.FLVF, Al.SPF and Al.MeF fractions had the lowest IC_50_ value, indicating that they contained the best inhibition of the enzyme. A significant correlation of antioxidant activity versus total phenolic contents and anticholinesterase potential versus total phenolic contents demonstrates that the phenolic compounds present in this plant specie are responsible for these activities which necessitate the further exploration of this plant for the isolation of bioactive compounds. Previous reports also demonstrates the presence of anticholinesterase compounds in various plant samples, which goes parallel with our current study [36,38-41].

## Conclusion

Based on the significant antioxidant and anticholinesterase potentials of *A. laciniata* it may be inferred that *A. laciniata* is a good source of anti-Alzheimer and neuro-protective natural compounds especially the polyphenolic compounds. Evidenced from the results, it may also be concluded that further isolation and characterization of this plant may lead to novel drug candidates against Alzheimer’s and neuro-degenerative disorders.

## Methods

### Plant collection, extractions and fractionation

The whole plant of *A. laciniata* was collected in May 2012 from ring road, Peshawar, Khyber Pukhtunkhwa (KPK), Pakistan. The plant was identified by botanical taxonomist Dr. Ali Hazrat Department of Botany, Shaheed Benazir Bhutto University, Sheringal Dir Upper, KPK, Pakistan and plant sample was deposited at the herbarium of the same University with voucher no. 1014.ZK.SBBU. The whole plant was subjected to shade drying. The dried plant was then grinded to fine powder. The powdered materials (6 Kg) were soaked in commercial grade methanol 85% (24 liters) for 15 days, as 1 Kg crude drug will be dissolved in 4 liter of methanol [42]. The crude extracts were filtered through filter paper and this process was repeated three times. The filtrates were concentrated at 40°C under reduced pressure, using a rotary evaporator (Heidolph, Germany), till a brownish crude extract (425 g) was obtained. The brownish Al.MeF (425 g, 7.08% of the whole powder) was dissolved in 1600 mL distilled water and was successively fractionated with (500 mL of each solvent, three times each) n-hexane, chloroform, and ethyl acetate [43]. The Al.MeF was fractionated by solvent–solvent partitioning to obtain four fractions, Al.EaF (7.0 gm, 1.65%), Al.CfF (26.0 gm, 6.1%), and Al.HxF (12.0 gm, 2.9%) and the residual Al.WtF (80.0 gm, 18.9%) fractions.

### Extraction of crude saponins

For the extraction of saponins, 20 g of powdered of plant sample were put into a conical flask; then 100 mL of 20% ethanol was added. The sample was then put into a water bath having temperature of 55°C for 4 hours with continuous stirring. The mixture obtained was filtered and the residue was extracted again with 200 mL ethanol (20%). The volume obtained was put into a water bath and the volume which was approximately 300 mL was concentrated to 40 mL which was then transferred into a separating funnel, adding 20 mL of diethyl ether into it with vigorous shaking. The separating funnel was kept for a while in a stand to obtain two layers; aqueous and organic. The aqueous layer obtained was recovered while the organic layer obtained was discarded and the process was repeated so that to achieve purification. After that 60 mL of *n*-butanol were added into the aqueous layer and put for a while. Then 10 mL of 5% NaCl solution was added to wash the combined *n*-butanol extract. The solution remained was evaporated by keeping in a hot water bath and then kept in an oven to get saponins in a dried form [44]. Near about 5.93 gm (29.65%) of Al.SPF were obtained.

### Extraction of crude flavonoids

For flavonoids extraction, standard procedure was followed. Briefly, 20 g powder of plant sample was extracted twice with 200 mL of 80% aqueous methanol at room temperature. The whole solution was then filtered through Whatman filter paper No-1. The filtrate was later transferred into crucibles. The sample was then put into a waterbath to evaporate until dryness. The dry residue left is the crude flavonoids [45]. This is then weighted to a constant weight (1.57 gm, 7.85% of the total weight).

### Chemicals and drugs

DPPH (CAS 1898-66-4 Sigma Aldrich CHEMIE GmbH USA), ABTS (CAS 30931-67-0 Sigma Aldrich USA), K_2_S_2_O_4_ (Riedel-de Haen Germany), Gallic acid and Folin Ciocalteu reagent (FCR) were purchased from Merck Co. (Germany). Enzymes including acetylcholinesterase *Electric eel* (type-VI-S, CAS 9000-81-1 Sigma-Aldrich GmbH USA ), butyrylcholinesterase equine serum Lyophilized (CAS 9001-08-5 Sigma-Aldrich GmbH USA), substrates acetylthiocholine iodide (CAS1866-15-5 Sigma-Aldrich UK), butyrylthiocholine Iodide CAS 2494-56-6 Sigma-Aldrich Switzerland), DTNB 5,5-dithio-bis-nitrobenzoic acid (CAS 69-78-3 Sigma-Aldrich Germany), Galanthamine hydrobromide Lycoris Sp. (CAS 1953-04-4 Sigma-Aldrich France) were used for enzyme inhibition study. For preparation of buffer, di-potassium hydrogen phosphate (K_2_HPO_4_), Potassium di-hydrogen phosphate (KH2PO_4_), potassium hydroxide used were of extra pure analytical grade, Hydrogen peroxide (H_2_O_2_).

### Total phenolic content

The hydroxyl groups on the phenols in the plant constituents give them radical scavenging ability which may be directly correlated with the antioxidant activity [46]. So for the determination of total phenolic contents in plant extract of *A. laciniata*, the procedure used by Kim *et al.,* [47] was followed. In this method, 1 mL of diluted extracts was added to 9 mL of distilled water, followed by addition of Folin-Ciocalteu’s reagent (1 mL) was added to it and properly shake. After 5 minutes 10 mL of 7% Na_2_CO_3_ solution was transferred into it and properly mixed. The mixture was diluted with 25 mL distilled water and mixed well. After 90 minutes the absorbance was measured at 750 nm using spectrophotometer (Thermo electron corporation, USA). Gallic acid standard curve was used as a reference to measure the total phenolic contents. The total phenolic contents were expressed as mg of Gallic Acid equivalent.

### Total flavonoid content

It has also been reported that the antioxidant activity of plant extract is attributed to the total flavonoid contents [48]. While for total flavonoid contents the procedure followed by Park *et al.,* [49] were pursued. In this 0.3 mL of the plant extracts, 0.15 mL of 0.5 M NaNO_2_, 3.4 mL of 30% methanol and 0.15 mL of 0.3 M AlCl_3_.6H_2_O were added to 10 mL test tube and was properly mixed. After 5 minutes, 1 mL of 1 M NaOH was added. Absorbance of mixture was measured at 506 nm. Rutin standard solution (0 to 100 mg/L) was used for determination of total flavonoids content and were expressed as mg of Rutin equivalent [50].

### DPPH radical scavenging activity

DPPH radical scavenging potential of plant samples were determined using procedure adopted by Brand-Williams *et al.,* [51]. For preparation of DPPH solution, 24 mg of DPPH was dissolved in 100 mL of methanol. Stock solutions (1 mg/mL) of plant extracts were prepared in methanol and were diluted to the concentrations of 125, 250, 500 and 1000 μg/mL. From these solutions, 1 mL was mixed with 1 mL of DPPH solution and was incubated at 23°C for 30 minutes. Absorbance was measured at 517 nm using UV Spectrophotometer (Thermo electron corporation, USA). Ascorbic acid was used for positive control, percent scavenging activity was calculated as; [(A_Blank_ – A_Sample_)/A_Blank_] × 100, where A_Blank_ represent absorbance of control and A_Sample_ is the absorbance of the plant extracts. Each experiment was done in triplicate and inhibition curves were constructed using the GraphPad prism program (GraphPad prism, San Diego, California, USA) and median inhibitory concentrations (IC_50_) values were determined.

### ABTS free radical scavenging assay

The antioxidant potential of *A. laciniata*, was also evaluated using 2, 2-azinobis [3-ethylbenzthiazoline]-6-sulfonic acid (ABTS) [52]. The assay is based on the capacity of antioxidants to scavenge ABTS^+^ radical cation causing a reduction in absorbance at 734 nm. In brief ABTS 7 mM and potassium persulphate (K_2_S_2_O_4_) 2.45 mM solutions were prepared and mixed. The resultant mixture was stored in dark at room temperature for 12–16 h to get dark colored solution containing ABTS radical cations. Prior to use, ABTS radical cation solution was diluted with Phosphate buffer (0.01 M) pH 7.4, to adjust an absorbance value of 0.70 at 734 nm. Radical scavenging ability of the fractions was analyzed by mixing 300 μl of test sample with 3.0 mL of ABTS solution in cuvette. The reduction in absorbance was measured spectrophotometrically, one minute after mixing the solutions and continued for six min. Ascorbic acid was used as positive control. The assay was repeated in triplicate and percentage inhibition was calculated using formula:

$$ \mathrm{Percent}\ \mathrm{scavenging}\kern0.37em \mathrm{effect}=\frac{\mathrm{control}\ \mathrm{absorbance}-\mathrm{sample}\ \mathrm{absorbance}}{\mathrm{control}\ \mathrm{absorbance}}\times 100. $$

The antioxidant effect was expressed in terms of percent inhibition and as EC_50_ (Extract concentration required for 50% reduction of ABTS radicals.

### Hydrogen peroxide scavenging activity

For the detection of hydrogen peroxide scavenging activity in the plant sample of *A. laciniata*, procedures of the Ruch *et al.,* [53] were followed. First prepared 2 mM solution of hydrogen peroxide in 50 mM phosphate buffer (pH = 7.4). Then 0.1 mL of various plant samples were taken in test tube and their volume were made 0.4 mL by addition of 50 mM phosphate buffer. Add 0.6 mL of hydrogen peroxide to it. After 10 minutes measure the absorbance of each sample at 230 nm against the blank [54]. Use the following equation for the calculation of hydrogen peroxide scavenging activity;

$$ \mathrm{Hydrogen}\ \mathrm{peroxide}\ \mathrm{scavenging}\ \mathrm{activity}=\left(1\hbox{-} \frac{\mathrm{absorbance}\ \mathrm{of}\ \mathrm{sample}}{\mathrm{absorbance}\ \mathrm{of}\ \mathrm{control}}\right)\times 100. $$

### Anticholinesterase assays

Acetylcholinesterase (AChE) from Electric eel and butyrylcholinesterase (BChE) from equine serum were used to explore the enzymes inhibitory potential of *A. laciniata*, its subsequent fractions, Al.FLVF and Al.SPF using Ellman’s assay [55]. The assay is based on the hydrolysis of acetylthiocholine iodide or butyrylthiocholine iodide by the respective enzymes and the formation of 5-thio-2-nitrobenzoate anion followed by complexation with DTNB to give yellow color compound which is detected with a spectrophotometer beside the reaction time.

### Preparation of solutions

Crude extract, subsequent fractions, Al.SPF and Al.FLVF were dissolved in phosphate buffer (0.1 M) in concentrations ranging from 125–1000 μg/mL. For the preparation of 0.1 M and 8.0 ± 0.1 PH phosphate buffer solution, K_2_HPO_4_ (17.4 g/L) and KH2PO_4_ (13.6 g/L) were prepared and were mixed in 94% and 6% ratio respectively. Finally potassium hydroxide was used to adjust PH. Then AChE (518 U/mg solid) and BChE (7–16 U/mg) were diluted in freshly prepared buffer pH 8.0 until a final concentration of 0.03 U/mL and 0.01 U/mL were obtained. Solutions of DTNB (0.0002273 M), ATchI and BTchI (0.0005 M) were prepared in distilled water and were kept in Eppendorf caps in the refrigerator. Galanthamine (Positive control) was dissolved in methanol and afore mentioned dilutions were prepared.

#### Spectroscopic analysis

For each assay, an enzyme solution of 5 μl was added to the cuvette, followed by addition of plant extract solution (205 μl), and finally DTNB reagent (5 μl). The solution mixture was maintained at 30°C for 15 min using water bath, and subsequently the substrate solution (5 μl) was added. A double beam spectrophotometer was used to measure the absorbance at 412 nm. Negative control contained all components apart from the plant extracts, whereas positive control galanthamine (10 μg/mL) was used in the assay as standard cholinesterase inhibitor. The absorbance along with the reaction time were taken for four minutes at 30°C and were repeated in triplicate. Finally the enzyme activity and enzyme inhibition by control and tested samples were calculated from the rate of absorption with change in time (V = ΔAbs /Δt) as follow;

$$ \begin{array}{l}\kern11.5em \mathrm{V}=\Delta \mathrm{Abs}/\Delta \mathrm{t}\\ {}\kern4em \%\ \mathrm{enzyme}\ \mathrm{activity}=\mathrm{V}/\mathrm{Vmax}\times 100\\ {}\%\ \mathrm{enzyme}\ \mathrm{inhibition}=100\hbox{-} \%\ \mathrm{enzyme}\ \mathrm{activity}\end{array} $$

### Estimation of IC_50_ values

Concentrations of the plant extract at which 50% of inhibition is observed (IC_50_) were calculated among the inhibition percentages against the extract concentrations via the Excel program.

### Statistical data analysis

All the assays were repeated in triplicate and vales were expressed as means ± Standard error means (SEM). Statistical analyses were performed by Two-Way analysis of variance (ANOVA), followed by Bonferroni post-tests. The difference was considered to be statistically significant when the *p* value was less than 0.05.
